# Progress of anti-tuberculosis drug targets and novel therapeutic strategies

**DOI:** 10.3389/fmicb.2025.1637254

**Published:** 2025-09-05

**Authors:** Yang Zhang, Ruiying Wu, Mingrui Sun, Xiaotian Li, Ren Fang, Jiayin Xing, Zhaoli Li, Yurong Wen, Ningning Song

**Affiliations:** ^1^Weifang Key Laboratory of Respiratory Tract Pathogens and Drug Therapy, School of Life Science and Technology, Shandong Second Medical University, Weifang, China; ^2^SAFE Pharmaceutical Technology Co., Ltd., Beijing, China; ^3^The First Affiliated Hospital of Xi'an Jiaotong University, Xi’an, China

**Keywords:** anti-tuberculosis drugs, tuberculosis treatment, *Mycobacterium tuberculosis*, drug target, novel therapeutics

## Abstract

Tuberculosis, a chronic infectious disease caused by *Mycobacterium tuberculosis* complex, has re-emerged as the leading cause of death worldwide as a single infectious agent. The increasing prevalence of multidrug-resistant tuberculosis and extensively drug-resistant tuberculosis poses a severe and growing threat to global health. Therefore, it is urgent to find new drug targets. Recently, significant advancements have been made in the research of drug targets and novel therapeutic strategies for tuberculosis. This review summarizes recent processes on anti-tuberculosis drug targets, such as cell wall synthesis, nucleic acid replication and transcription, energy metabolism, and ferroptosis. Furthermore, this review summarizes the research progress of three innovative tuberculosis treatment strategies, including antimicrobial peptides, host-directed therapies, and nanoparticle-based drug delivery systems, aiming to provide a theoretical foundation and new research perspectives for the clinical development of new drugs.

## 1 Introduction

Tuberculosis (TB) is a communicable disease primarily caused by *Mycobacterium tuberculosis* complex (MTBC). Mtb primarily affects the lungs, which mainly spreads through droplets, particularly coughing, sneezing, or spitting (Kaufmann and Winau, 2005). According to the 2024 World Health Organization (WHO) Global Tuberculosis Report, there were approximately 10.8 million new cases of TB and 1.25 million deaths. TB has surpassed coronavirus disease 2019 (COVID-19) to re-emerge as the world’s leading cause of death as a single infectious agent (World Health Organization, 2024). In clinical treatment, different TB treatment strategies are adopted due to variations in drug sensitivity. Drug-sensitive tuberculosis (DS-TB) typically adopts a standardized 6-month short-course chemotherapy regimen, consisting of a 2-month intensive phase with a combination of isoniazid (H), rifampicin (R), pyrazinamide (Z), and ethambutol (E), followed by a 4-month continuation phase with H and R for sustained bactericidal treatment. However, drug-resistant tuberculosis (DR-TB), particularly multidrug-resistant tuberculosis (MDR-TB) and extensively drug-resistant tuberculosis (XDR-TB), requires individualized regimens with second-line drugs like bedaquiline and linezolid. Common DR-TB treatment drugs are shown in Table 1. It is worth noting that prolonged TB treatment can cause various adverse effects such as hepatotoxicity, hyperuricemia, ototoxicity, and neuropsychiatric manifestations (Alsayed and Gunosewoyo, 2023; Trajman et al., 2025). Therefore, drug resistance poses a serious challenge to TB prevention and treatment efficacy.

**Table 1:** Common drug-resistant TB treatment drugs.

Classification of drugs	Name of drugs
Group A (First-choice drugs)	Moxifloxacin, Levofloxacin, Bedaquiline, Linezolid
Group B (Alternative drugs)	Clofazimine, Cycloserine
Group C (Supplementary drugs)	Ethambutol, Delamanid, Pyrazinamide, Imipenem/Cilastatin, Macrolide

The drug resistance of Mtb includes two major types: intrinsic resistance and acquired resistance. Mtb possesses an abnormally thick and lipid-rich cell wall, which restricts the entry of most hydrophilic drugs and is a key factor contributing to intrinsic drug resistance. Mtb can form biofilms which play a key role in blocking the drug penetration. This creates a protective environment that greatly increases bacterial tolerance to antibiotics. On the other hand, chromosomal gene mutations are the main mechanism of acquired drug resistance (Rabaan et al., 2022). For example, mutations in the *rpoB* gene prevent the effective interaction between drugs and RNA polymerase (RNAP), leading to rifampicin resistance (Jose Vadakunnel et al., 2025). The most common mutation is *rpoB* Ser531Leu, which is associated with high-level resistance. Other frequent mutations include Asp516Val and His526Tyr, both of which confer moderate to high levels of resistance (Sinha et al., 2020). His526Tyr, His526Asp, and His526Leu are particularly prevalent among resistant strains and are considered key hotspot mutations (Li et al., 2021). Although Val170Phe and Ile491Phe occur outside the rifampicin resistance-determining region, studies have reported that they can also confer rifampicin resistance (Ma et al., 2021). Mutations in *katG and inhA* cause isoniazid resistance (Nono et al., 2025). All mutations in the *katG* gene are associated with high-level resistance to INH, with the most common mutation being *katG* S315T1 (Singh et al., 2025). In contrast, mutations in the *inhA* gene typically confer low-level resistance, where high doses of INH may still be effective. A frequently observed mutation in this region is MUT1 (C-15 T) (World Health Organization, 2023). When both S315T1 and C-15 T mutations are present simultaneously, they can result in a significantly higher level of resistance, potentially exceeding the clinically achievable serum concentration of INH (≥19 mg/L), rendering the drug completely ineffective (Lempens et al., 2018). Mutations in *gyrA* reduce the binding affinity of drugs with their targets, resulting in fluoroquinolone resistance (Dixit et al., 2023). Among these, Ala90Val and Asp94 (Gly/Ala/His/Asn) are the most commonly observed mutations associated with fluoroquinolone resistance (Von Groll et al., 2009). Notably, T80A and A90G mutations have been reported to partially restore fluoroquinolone susceptibility when present alongside resistance-associated mutations such as A90E or D94N (Pantel et al., 2016). Additionally, mutations in the *embB* gene affect the efficacy of ethambutol (Li et al., 2025). Due to the long-term clinical use and the limitation of existing drugs, it is urgent to discover novel drug targets and develop new antimicrobials for TB therapy. The target proteins, binding sites, and critical residues of first-line anti-tuberculosis drugs are summarized in Table 2.

**Table 2:** Binding sites and critical residues of anti-TB drugs.

Drugs	Target protein	Key binding sites	Critical residues	Reference
Isoniazid	InhA	NADH-binding site and INH adduct	Ser94, Tyr158, Ile21	Zhang et al. (2022) and Davoodi et al. (2023)
Rifampicin	RpoB	Rifampicin-binding pocket in RNA polymerase	Ser531, His526, Asp516	Molodtsov et al. (2017) and Petry et al. (2020)
Ethambutol	EmbB	Arabinosyl transferase active site	Met306	Bwalya et al. (2022)
Fluoroquinolones	GyrA	Quinolone Resistance-Determining Region	Ala90, Ser91, Asp94, Gly88	Kabir et al. (2020) and Sekiguchi et al. (2011)

**Figure 1:**
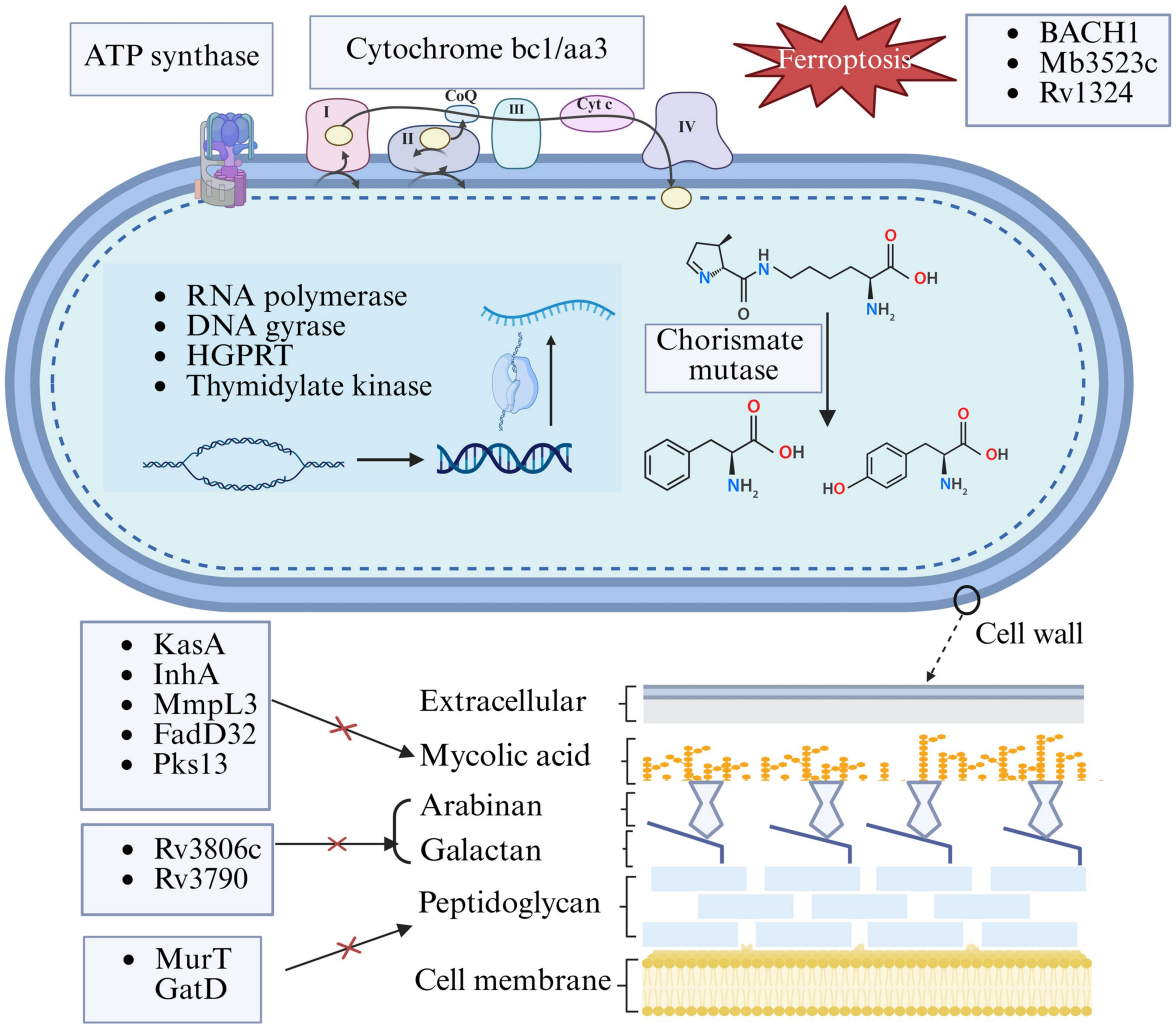
Key drug targets in *Mycobacterium tuberculosis*. This figure illustrates key molecular targets for current and investigational anti-tuberculosis drugs within *Mycobacterium tuberculosis*. The targets are categorized based on their roles in essential cellular processes, including cell wall synthesis, DNA replication and transcription, energy metabolism, and Ferroptosis. The figure highlights both established and emerging pathways exploited for therapeutic intervention, reflecting recent advances in drug discovery against *Mycobacterium tuberculosis*.

Recently, the molecular mechanisms of pathogenicity and drug resistance of Mtb have been deciphered, which further deepened the study of traditional drug targets. Meanwhile, the discovery of emerging targets, such as the phosphoribosyltransferase Rv3806c and the key lipid metabolism enzyme Pks13, has provided novel opportunities for drug design. Additionally, the exploration of novel therapeutic strategies is expanding in multiple directions. The development of antimicrobial peptides, host-directed therapies that modulate the immune response, and nanocarrier drug delivery systems that enhance drug penetration offers new opportunities for TB treatment. This review summarizes the new discoveries in classical targets and the development of core targets/inhibitors within the key Mtb pathways, including cell wall synthesis, energy metabolism, nucleic acid processes, and ferroptosis (Figure 1). This review aims to accelerate the development of safer, more efficient, and shorter-course next-generation anti-TB drugs. It also explores the potential and challenges of innovative therapeutic strategies from the perspectives of drug delivery and immune modulation, providing insights for TB prevention and treatment.

## 2 Anti-tuberculosis drug targets

### 2.1 Key enzymes in cell wall synthesis

The cell wall of Mtb with complex structure not only maintains cell integrity but also enables the bacterium to adapt to the host environment, which plays a crucial role in chronic infection and pathogenesis. The cell wall of Mtb primarily consists of three components: peptidoglycan (PG), arabinogalactan (AG), and mycolic acids (MAs) (Abdelaziz et al., 2021). Targeting key catalytic factors involved in the biosynthesis of the Mtb cell wall is a promising approach for developing new anti-TB drugs. For example, disrupting the function of core proteins encoded by *Rv3806c* and *Rv3790* in the AG synthesis pathway, or inhibiting key proteins such as *β*-ketoacyl-acyl carrier protein synthase I, enoyl-ACP reductase, and the mycobacterial membrane protein Large 3 in the MAs biosynthesis pathway, has been shown to effectively inhibit Mtb growth. Additionally, targeting the essential proteins MurT and GatD in PG synthesis, along with resistance-associated *β*-lactamase, provides effective strategies to weaken the cell wall and enhance antibiotic efficacy. These proteins are vital for maintaining the stability of the bacterial cell wall and have emerged as promising targets for the development of novel anti-TB therapies (Diab et al., 2025).

#### 2.1.1 Rv3806c

Rv3806c is a membrane-bound phosphoribosyltransferase (PRTase) that catalyzes the transfer of the pentose phosphate group from ribose phosphate pyrophosphate to decylisopentenyl phosphate to generate decylisopentenyl-1-phosphate-β-ribosyl-5-phosphate (DPPR). DPPR is the precursor of decylisopentenyl phosphate arabinose (DPA), the exclusive arabinosyl donor known to be involved in the synthesis of cell wall precursors. Consequently, the Rv3806c protein has emerged as a critical therapeutic target for the development of novel antitubercular agents, owing to its indispensable role in mycobacterial survival and its conserved structural features (He et al., 2015).

Ethambutol (EMB) is a widely used anti-TB drug in clinical practice. Its target is a glycosyltransferase encoded by the *embCAB* operon. The EmbCAB protein catalyzes the transfer of arabinose residues, facilitating the formation of arabinoglycan chains that are essential for AG synthesis in cell wall of Mtb. This process is critical for maintaining the structural integrity and growth of the cell wall, making EmbCAB proteins a key target for the development of novel anti-TB drugs (Sun et al., 2017; Xiang et al., 2021). Mutations in the *embB* leads to resistance to EMB, 159 MDR-TB isolates were obtained from 159 patients with pulmonary TB in China. Research has shown that 95.6% (109/114 isolates) of EMB-resistant isolates harbored at least one mutation within the regions associated with EMB resistance. Most mutations were in *embB*, particularly between amino acid positions 300 and 500, and in the *embC*–*embA* intergenic region. The most frequently mutated residues were Met306, Gly406, and Gln497, which were identified in 87 EMB-resistant isolates and 2 EMB-susceptible isolates (Li et al., 2025). Additionally, *rv3806c* mutations lead to DPA overexpressed, which competes with EMB for binding to EmbCAB proteins, thereby contributing to increased drug resistance. Tulyaprawat et al. (2019), isolated both EMB-sensitive and EMB-resistant Mtb strains in clinical settings with mutations in the *ubiA* gene. It was shown that there was a strong association with 100% specificity between *ubiA* mutations and stronger EMB resistance, which suggested it could serve as a marker for EMB resistance.

Additionally, Gao et al. (2024), reported the cryo-electron microscopy-derived three-dimensional structure of Rv3806c in both the donor- and acceptor-bound states, thereby elucidating the molecular mechanism by which this protein catalyzes ribose phosphate transfer at the bacterial plasma membrane. Rv3806c closes the active site through a conformational change and promotes the transfer of ribose phosphate from 5-Phospho-*α*-ribosyl-1-pyrophosphate (PRPP) to Decaprenyl phosphate (DP) to generate DPPR. The elevated rate of DPPR synthesis results in EMB resistance. This process follows an inversion mechanism whereby the C_1_ conformation of the ribose is changed from α to *β*. Lys28 is involved in the binding between DP and PRPP, which is essential for the catalytic reaction. Tyr70 and Tyr138 participate in the stabilization of the reaction transition state. Asp77 and Asn73 stabilize the pyrophosphate moiety of PRPP by coordinating with Mg^2+^ ions. Gln135 forms hydrogen bonds with the ribose moiety of PRPP to ensure the correct localization of the substrate. Therefore, Rv3806c is a crucial target for anti-TB therapy, offering potential strategies for novel drug discovery and addressing resistance to current treatments.

#### 2.1.2 DprE1(Rv3790)

DprE1, as a key enzyme in the production of AG precursor, plays an important role in mycobacterial cell wall synthesis (El Haddoumi et al., 2024; Delgado et al., 2024). It is shown that drugs targeting DprE1 can disrupt cell wall integrity to make bacterial cell death without entering the cytoplasm to carry out effects (Brecik et al., 2015). As a result, DprE1 is considered a highly promising target for the development of novel anti-TB drugs. Benzothiazinones (BTZs) were the first inhibitors of DprE1, serving as the foundation for the continuous optimization and design of both covalent and non-covalent DprE1 inhibitors. However, none of these have been approved for clinical use (Makarov et al., 2009). Recently, novel DprE1 inhibitors have been identified through high-throughput screening and designed using artificial intelligence and computer-aided drug design (AI/CADD) tools. Yang F. et al. (2024) and Yang L. et al. (2024), identified a series of novel N-(1-(6-oxo-1,6-dihydropyrimidin)-pyrazole) acetamide derivatives with significant activity against Mtb via structure-based virtual screening and computational-guided design. Among these, the compounds LK-60 and LK-75 effectively inhibited the growth of Mtb and the activity is significantly superior to the phase II candidate TBA-7371. Currently, four DprE1 inhibitors are in clinical development, BTZ-043 and PBTZ-169 are suicide inhibitors that irreversibly inhibit DprE1 by forming a semimercaptal bond with the active-site cysteine (Cys387) via nitro group. TBA-7371 and OPC-167832 bind reversibly and stably to the active site of the DprE1 through multiple non-covalent interactions, such as hydrogen bonds and hydrophobic forces, effectively inhibiting the cell wall synthesis of Mtb (Alsayed and Gunosewoyo, 2023). Heinrich et al. (2025), further evaluated the safety, bactericidal activity and pharmacokinetics of BTZ-043. Bioequivalence tests showed that BTZ-043 had little effect on the metabolism of caffeine and digoxin, indicating that BTZ-043 can be used in combination with these drugs. BTZ-043 showed good safety and bactericidal activity, and the 1,000 mg dose in combination with standard breakfast may be the optimal therapeutic regimen. With its novel mechanism of DprE1 inhibition and enhanced tissue penetration, BTZ-043 holds great potential to shorten the duration of TB treatment. Subsequent studies will focus on how to optimize combination therapies and drug resistance.

#### 2.1.3 *β*-Ketoacyl-ACP synthase I

MAs is a crucial component of the cell wall in Mtb, essential for stabilizing its structure and maintaining its density. MAs is synthesized through the joint catalysis of two fatty acid synthases: one resembling the multifunctional fatty acid synthase (FAS-I) found in eukaryotes or higher prokaryotes, and the other similar to the fatty acid synthase (FAS-II) found in plants or bacteria. β-ketoacyl-ACP synthase I (KasA) in the FAS-II system is considered to be a potential drug target for anti-TB therapy (Shinde and Suvarna, 2022; Rudraraju et al., 2022). Thiacetazone (TLM) and its derivatives are recognized as inhibitors of KasA enzyme activity. However, TLM analogs demonstrate limited efficacy in inhibiting the KasA protein (Kremer et al., 2000; Kapilashrami et al., 2013). It has been found that isoxazole sulfonamide derivatives have been identified as potential inhibitors of KasA enzyme activity, although the precise mechanism remains unknown (Bajad et al., 2022; Inoyama et al., 2020). Based on its structure, Adewumi et al. (2023), initially screened 817 anti-*Mycobacterium* compounds from the ZINCPharmer chemical database. ZINCPharmer is a virtual screening tool based on pharmacophore models and built on the ZINC database, which contains millions of commercially available small molecules. It allows researchers to quickly identify potential bioactive compounds based on 3D pharmacophore features, aiding early-stage drug discovery.^1^ These compounds were subjected to comprehensive computer-aided drug design screening to identify six potential KasA inhibitors. Compared to the standard inhibitor thiacetazone, these candidates exhibited a higher binding affinity for the KasA active site. These findings provide new insights into the development of TB drugs, particularly for targeted therapy against KasA.

KasA catalyzes the elongation of acyl chains, however, the mechanism by which KasA selectively recognizes and excludes shorter acyl molecules remains unclear (Luckner et al., 2009; Lee and Engels, 2014). Studies have demonstrated that when the C_18_ receptor completely binds to KasA, interactions between the receptor’s terminal and its surrounding environment (including Glu120 and adjacent regions) trigger a conformational change in Phe404, causing a transition from a closed to an open state in the neighboring subunit. This change activates the catalytic residues in that subunit, suggesting the presence of positive cooperativity in KasA. The induced cooperative signal is initiated by the terminal of the C_18_ receptor, while shorter receptors, such as C_16_, fail to interact with Glu120 and Tyr126’, thereby hindering the activation of this signal. These findings elucidate the molecular mechanism by which KasA is selectively activated only by substrates of the appropriate length (Lee, 2024).

#### 2.1.4 Enoyl-acyl carrier protein reductase

InhA, an enoyl-acyl carrier protein reductase, is a key enzyme in the FAS-II system, driving the synthesis of MAs essential for mycobacterial cell wall formation. It is also the primary target of INH which requires the activation by the enzyme KatG. However, due to mutations in *KatG*, mycobacteria have gradually developed increased resistance to INH recently. In eastern Uttar Pradesh, India, 6.57% of sputum-positive TB samples exhibited resistance to INH. Among these resistant cases, the majority (77.9%) exhibited high-level resistance, predominantly associated with the *katG* MUT1 (S315T1) mutation, while the remaining 22.1% displayed low-level resistance associated with *inhA* MUT1 (C-15 T) mutation. This study confirms the predominant role of *katG* mutation in high-level and *inhA* mutation in low-level INH resistance in this population (Singh et al., 2025). In a study of 500 INH-resistant Mtb isolates from Cameroon, 60.2% carried *katG* mutations, predominantly the S315T variant (Nono et al., 2025). These findings highlight that *katG* S315T acts as a primary marker for INH resistance. Therefore, in order to overcome resistance, it is essential to design InhA inhibitors that do not require prior activation, such as triclosan derivatives (Vosátka et al., 2018; Chetty et al., 2021; Shekhar et al., 2023), coumarin derivatives (Batran et al., 2024; Kassem et al., 2014), 1,8-naphthyridine-3-carbonitrile analogs (Khetmalis et al., 2024), sulfonylhydrazone derivatives (Teneva et al., 2024; Angelova et al., 2022), all of which are classified as non-prodrug-type InhA inhibitors.

#### 2.1.5 MurT and GatD

PG undergoes two distinct modifications: N-glycosylation of wall acids and amidation of D-glutamic acid. N-glycosylation enhances the immunogenicity of the Mtb cell wall without contributing to its pathogenicity (Hansen et al., 2014). Meanwhile, amidation of D-glutamic acid facilitates the cross-linking of PG precursors. In this process, MurT and GatD catalyze the amidation reaction. The extensive depletion of MurT and GatD increases the permeability of the cell wall, leading to a reduced sensitivity to *β*-lactam antibiotics (Catalão et al., 2019; Shaku et al., 2023). Silveiro et al. (2023), utilized CRISPR interference (CRISPRi) technology to knockout genes encoding PG modification enzymes (*namH* and *murT*/*gatD*) of *Mycobacterium smegmatis* (Ms). The results demonstrated that PG modifications play a key role in β-lactam resistance and survival of the pathogen within the host, highlighting the potential of these enzymes as therapeutic targets for TB treatment.

The characteristic modifications of mycobacterial PG promote antibiotic resistance and survival of Mtb within host macrophages. Inhibiting the enzymes responsible for these peptidoglycan modifications may serve as a strategy against TB and play a key role in shortening the duration of TB treatment in the future.

#### 2.1.6 MmpL3

MmpL3 is a transmembrane protein belonging to the MmpL family, responsible for transporting MAs precursors from the cytoplasm to the cell wall. Dysfunction of MmpL3 leads to cell wall synthesis disruption, thereby inhibiting bacterial growth. As a key protein in the cell wall biosynthesis of Mtb, MmpL3 is an important target in anti-TB therapy (Degiacomi et al., 2017; Williams and Abramovitch, 2023). Recently several small molecule inhibitors targeting MmpL3 have been discovered. SQ109 is one of the MmpL3 inhibitors and is currently in Phase III clinical trials. However, due to degradation by cytochrome P450 enzymes CYP3A4 and CYP2C19, the inhibitor has a short half-life and low oral bioavailability in the host (Carbone et al., 2023). AU1235 demonstrates strong bactericidal activity both *in vitro* and *in vivo* with low cytotoxicity. Currently, it remains in preclinical studies and requires further validation of its safety and efficacy (Luo et al., 2021). The unique chemical structure of BM212 with a bifunctional aryl-pyrrole framework provides potent bactericidal activity and high selectivity, making it one of the important candidates for anti-TB drug development (Supplementary Figure 1). To enhance their inhibitory potency, Vasudevan et al. (2023), designed and synthesized several bifunctional aryl-pyrrole silicon analogs related to BM212. Through Alamar Blue assays, it was found that most of silicon-containing compounds were more effective against Mtb than BM212, offering new directions for the further development of anti-TB drugs. TBI-166, derived from a clofazimine analog, exhibits more potent anti-TB activity than clofazimine. It is currently undergoing Phase IIa clinical trials in China. Furthermore, the combination of TBI-166 with bedaquiline and pyrazinamide has been recommended for further investigation in Phase IIb clinical trials (Ding et al., 2022).

Williams et al. (2024), discovered a novel MmpL3 inhibitor, MSU-43085, which inhibits Mtb, *Mycobacterium abscessus* (MAB), and *Mycobacterium avium* (MAC) both *in vivo* and *in vitro*. Pharmacokinetic studies show that MSU-43085 has high bioavailability and a short half-life. Efficacy studies *in vivo* found that MSU-43085 inhibited Mtb in an acute mouse TB infection model, but lacked activity in a chronic mouse TB infection model. These results indicate that MSU-43085 is a potent inhibitor of Mtb and MAB, showing strong therapeutic potential.

#### 2.1.7 FadD32

Based on sequence analysis, FadD proteins can be classified into two categories: fatty acyl-AMP ligases (FAALs) and fatty acyl-CoA ligases (FACLs). FadD32, a member of the FAAL family, is one of the most extensively studied FadD enzymes in Mtb. In the presence of ATP, it activates long-chain fatty acids to form acyl-adenylate, which is then transferred to multifunctional polyketide synthases for further chain elongation. Therefore, it is crucial for Mtb survival and plays a key role in the biosynthesis of MAs (Alsayed et al., 2019). The crystal structure analysis provides a foundation for structure-based drug development (Chen et al., 2022). Rani et al. (2025), identified M1, a compound with an isoxazole scaffold, as a selective inhibitor of Mtb through virtual screening. When combined with rifampicin and isoniazid, M1 exhibits enhanced inhibition of Mtb, demonstrating greater efficacy than the standard TB treatment regimen. The inhibition mechanism is characterized by non-competitive inhibition of Mtb FadD32 with lauric acid and partial non-competitive inhibition with ATP. M1 is an effective chemical scaffold with the potential to inhibit multiple FadD family enzymes in Mtb, showing promise as a candidate for TB treatment.

#### 2.1.8 Pks13

Polyketide Synthase 13 (Pks13) plays a crucial role in the biosynthesis of MAs, which are essential for maintaining the integrity of the Mtb cell wall. As a result, Pks13 is an effective target to inhibit this pathway (Khola et al., 2024). Zhang et al. (2025), employed a structure-based drug design approach to synthesize and evaluate a series of compounds with anti-Mtb Pks13 activity. Among them, compounds 31, 41, 43, and 44 showed potent inhibitory activity against Mtb H37Rv, along with improved metabolic stability. Compound 44, with favorable bioavailability, shows promising potential as an anti-TB drug. Liu et al. (2025), discovered a compound named BMVC-8C3O that effectively inhibits the activity of Pks13. This compound demonstrates activity against both MDR-Mtb and XDR-Mtb, while exhibiting low cytotoxicity. BMVC-8C3O binds to Pks13 in a distinctive manner, competitively inhibiting its enzymatic activity by forming hydrogen bond interactions with key amino acids in Pks13-TE, such as Asn1640, Ser1533, Tyr1674, and Phe1670, thereby exerting antimicrobial effects (Supplementary Figure 2). BMVC-8C3O features a novel structure, distinct from known Pks13 inhibitors such as thienyl, benzofuran, coumarin, flavonoid, and *β*-lactam scaffolds. This provides new insights into the design and optimization of Pks13 inhibitors.

Johnston et al. (2024), report the molecular structure of the catalytic core domains of Mtb Pks13 (Mt-Pks13) with 3.4 Å resolution through transmission cryo-electron microscopy. The assembly state of monomer and dimer for Mt-Pks13 is PH-dependent, and the comparison with the structure of Ms- Pks13 shows that Mt-P ks13 has conformational flexibility. The availability of diverse structures of this promising target for antimycobacterial therapy gives options for computer-aided drug discovery or design.

#### 2.1.9 *β*-lactamase

Although *β*-lactam antibiotics are the first-choice for treating various bacterial infections, they have not been widely used for TB treatment over the past decades. This is mainly because Mtb produces a potent *β*-lactamase named BlaC that rapidly hydrolyzes most β-lactam drugs (Kumar et al., 2022). The cell wall of Mtb contains a large amount of MAs and a complex lipid layer, forming a highly hydrophobic and impermeable barrier. This barrier effect makes it difficult for many antibiotics to penetrate the cell and exert their effects. PG cross-linking in Mtb mainly depends on L, D-transpeptidases rather than D, D-transpeptidases, which are common in typical bacteria. Most conventional *β*-lactam antibiotics primarily target D, D-transpeptidases and exhibit very weak activity against L, D-transpeptidases. Therefore, it is difficult to inhibit the cell wall synthesis of Mtb even if some of the drugs penetrate the cell wall (Cordillot et al., 2013). Hugonnet et al. (2009), showed that combining meropenem with clavulanate effectively inhibits BlaC, thereby restoring the bactericidal activity of *β*-lactam antibiotics against XDR-Mtb, primarily through inhibition of L, D-transpeptidases. This signifies the renewed attention to β-lactam antibiotics, especially carbapenems, in the treatment of DR-TB. It was the first to clearly propose a novel strategy for treating TB using a combination of carbapenems and β-lactamase inhibitors (BLIs).

Recent studies have focused on specific BLIs that can restore β-lactamase activity against drug-resistant TB. Clavulanic acid, an oral β-lactamase inhibitor, is the most commonly used for TB treatment, typically in combination with imipenem and meropenem. This is due to its irreversible inhibition of BlaC, which enables the effective killing of Mtb by *β*-lactam drugs (Shi et al., 2025). Durlobactam is a β-lactamase inhibitor, classified as a diazabicyclooctane compound, which inhibits β-lactamase activity to protect β-lactam antibiotics from degradation. It covalently binds to the serine residue at the active site of *β*-lactamase, thereby inhibiting enzyme activity and preventing the degradation of β-lactam antibiotics (Nantongo et al., 2024). When combined with meropenem or imipenem, durlobactam significantly enhances their bactericidal activity. Although β-lactam antibiotics are not currently used in TB treatment, their combination with BLIs represents a promising strategy for treating MDR-TB (Shin et al., 2025; Longo et al., 2024). Besides the inhibitors discussed above, agents such as avibactam and sulbactam are also used in combination therapies to enhance the activity of β-lactam antibiotics against resistant bacterial strains (Srivastava et al., 2021; Malla et al., 2023).

Facing the challenges of strong drug resistance and difficulties in in MDR-TB and XDR-TB treatment, the combination of BLIs with β-lactam antibiotics can expand therapeutic options, shorten treatment duration, and improve efficacy. These drugs are safe and well tolerated, and their combination application can reduce the dose and toxic side effects of traditional drugs, which has important clinical application value. Future research should focus on developing broader-spectrum and more effective inhibitors, optimizing combination regimens, and systematically evaluating their clinical efficacy and safety to advance their application in TB treatment.

### 2.2 Drug targets related to replication and transcription of nucleic acids

#### 2.2.1 RNA polymerase

Rifampicin is a broad-spectrum antibiotic that prevents bacterial RNA synthesis from survive by binding to the β-subunit of RNA polymerase (RNAP), preventing its interaction with DNA. As the core enzyme in the transcription process, RNAP is a crucial target for the development of anti-TB drugs (Campbell et al., 2001). The resistance to rifampicin primarily arises from mutations in the *rpoB* gene, which encodes the β-subunit of RNAP (Jose Vadakunnel et al., 2025). Zaw et al. (2018), found that the most common mutations occur at codon positions 516, 526, and 531, which can alter the conformation of RNAP, reduce its affinity for rifampicin, and ultimately prevent the drug from effectively inhibiting RNA synthesis. By analyzing the crystal structure of the RNA polymerase–rifampicin complex, key interactions between the drug and mutation sites can be identified, enabling the development of rifampicin analogs that overcome known resistance mutations. Rajeswaran et al. (2022), applied structure-based drug design to synthesize benzoxazinorifamycins (bxRIFs), congeners of the clinical candidate rifalazil. The structure of the bxRIF is presented in Supplementary Figure 3. Although this compound did not exhibit sufficient activity against Mtb RNAP for clinical application in the treatment of DR-TB, it demonstrated favorable pharmacological properties. These findings underscore the potential for further optimization of rifamycin derivatives to enhance their efficacy against Mtb. Additionally, the development of a scalable synthesis method for bxRIFs supports their feasibility for advancement into both preclinical and clinical research stages.

Clinical studies have shown that more than 90% of rifampicin-resistant Mtb carry mutations in the RRDR region of the *rpoB* gene. Research targeting RNAP not only helps to deeply understand the key binding mechanism between drugs and mutation sites, but also reveals which mutations lead to high levels of resistance, thus providing a scientific basis for designing drugs through structure optimization in order to avoid the effects of drug resistance. The new generation of RNAP inhibitors is expected to break through the limitations of traditional rifampicin-based drugs, target *rpoB* mutant strains, restore drug sensitivity, and provide a new solution for the treatment of multidrug-resistant and extensively DR-TB.

#### 2.2.2 DNA gyrase

DNA gyrase in Mtb is a type II topoisomerase involved in DNA replication, transcription, and the regulation of DNA supercoiling, consisting of two GyrA and two GyrB subunits. As this enzyme is absent in humans, it represents a crucial target for the development of anti-TB drugs (Crunkhorn, 2022). Quinolone drugs can inhibit DNA gyrase and are used to treat TB. In recent years, fluoroquinolone resistance has largely resulted from mutations in the GyrA subunit, Dixit et al. (2023), examined the relationship between fluoroquinolone antibiotic resistance mutations and treatment outcome. A particular focus of this study is the effect of mutations in the *gyrA* gene on fluoroquinolone resistance. It was found that the most common mutation was D94G (*gyrA* MUT3C, 44/150, 66%) among the *gyrA* resistance mutations. Drug research and target screening turn to GyrB subunits (Spencer and Panda, 2023). The indole derivative G24, which inhibits the ATPase activity of GyrB, demonstrates superior activity against Mtb compared to the well-known DNA gyrase inhibitor novobiocin. For the moment, Novobiocin is the only clinically approved DNA gyrase ATPase inhibitor (Pakamwong et al., 2022; Burlison et al., 2006). Pakamwong et al. (2024), used G24 as a template and carried out virtual screening to identify and characterize several potential inhibitors from the Specs compound library. Compounds 8, 11, and 14 were found to inhibit DNA gyrase activity, exhibiting 5-fold, 2-fold, and 16-fold higher activity than novobiocin, respectively. These findings provide strong theoretical support for the development of novel ATPase inhibitors.

#### 2.2.3 Hypoxanthine-guanine phosphoribosyltransferase

Hypoxanthine-guanine phosphoribosyltransferase (HGPRT) is a crucial enzyme in purine metabolism, responsible for converting hypoxanthine and guanine into their respective nucleotides. A deficiency in HGPRT disrupts the synthesis of purine nucleotides, impairing the production of DNA and RNA (Eng et al., 2018). Knejzlík et al. (2020), confirmed that HGPRT is the main guanine and hypoxanthine salvage enzyme in Ms but is not necessary under normal growth conditions. Research indicates that prodrugs derived from ANP-based inhibitors designed for the highly similar Mtb HGPRT do not target Ms. HGPRT but display antimicrobial activity against Ms. This finding raises the questions about the selectivity of known Mtb HGPRT-targeted compounds in bacterial cells.

#### 2.2.4 Thymidylate kinase

Thymidylate kinase (TMPK) is a pivotal enzyme in DNA biosynthesis, primarily responsible for catalyzing the phosphorylation of thymidine monophosphate (dTMP). The low sequence homology (22%) of TMPK between Mtb and human make it an attractive target for the development of novel anti-TB therapeutics (Jian et al., 2020; Sukumar et al., 2020). Venugopala et al. (2020), reported several tetrahydropyrimidinone derivatives, including pyrimidinone and pyrimidinthione. These compounds, as potential thymidylate kinase inhibitors, demonstrated antibacterial activity against Ms. and, particularly, pyrimidinone 1a and pyrimidinthione 2a effectively inhibited the growth of Mtb. Notably, compound 2a was observed to exert modest activity at 128 μg/mL against Mtb strains with cross-resistance to rifampicin and isoniazid. These findings suggest that compounds 1a and 2a could serve as potential anti-TB drugs, offering new insights for future drug design and research.

### 2.3 Drug targets related to energy metabolism

#### 2.3.1 ATP synthase

ATP synthase is not only a crucial drug target in Mtb, but also the primary target of bedaquiline used in MDR-TB treatment. It has been reported that amiloride derivatives can simultaneously inhibit cytochrome bd oxidase and F₁Fo-ATP synthase, demonstrating potential for development as anti-TB drugs (Hards et al., 2022). Adolph et al. (2024), discovered a compound called BB2-50F-6-derivative, which exhibits dual targeting activity. It inhibits both F₁Fo-ATP synthase and succinate dehydrogenase, effectively suppressing the growth of Mtb. When used in combination with other TB drugs, BB2-50F demonstrates higher bactericidal activity and enhance the effectiveness of treatment. Concurrently, the inhibitor targeting the ATP synthase complex named sudapyridine is currently under investigation for its efficacy against rifampicin-resistant TB, and has already entered phase III clinical trials (Yao et al., 2022; Yu et al., 2024).

#### 2.3.2 The cytochrome bc1: aa3

The cytochrome bc1: aa3 complex is responsible for transferring electrons from coenzyme Q to cytochrome c, and subsequently to oxygen, catalyzing the reduction of oxygen to form water. At the same time, it pumps protons to maintain the proton gradient, driving ATP synthesis. The key proteins have become the critical targets for anti-TB drug development (Sindhu and Debnath, 2022; Bajeli et al., 2020). Telacebec (Q203) is a potent drug candidate currently under clinical development for DR-TB treatment. The compound inhibits the respiratory chain of Mycobacterium by disrupting the function of the cytochrome b subunit (QcrB), thereby blocking the oxidative phosphorylation process. Currently, Q203 is in phase II clinical trials and has shown significant promise as one of the potential key drugs for future TB treatment (Pethe et al., 2013; Kang et al., 2014). Nguyen et al. (2022), investigated the interactions between Q203 and several anti-TB drugs or candidates, including bedaquiline, PBTZ169, PA-824, OPC-67683, SQ109, isoniazid, rifampicin, streptomycin, and linezolid. They found there were no antagonistic interactions between Q203 and the tested drugs, with most interactions being synergistic. Among these, the combination of Q203 and PBTZ169 was the most effective, showing stronger inhibition for Mtb compared to Q203 alone. This finding provides a new strategy for the development of anti-TB drugs.

#### 2.3.3 Chorismate mutase

Mtb is unable to directly obtain adequate phenylalanine and tyrosine from the host and therefore depends on its endogenous shikimate pathway for biosynthesis. Chorismate mutase (CM) acts in the shikimate pathway, catalyzing the conversion of chorismate to prephenate, which then enters the phenylalanine/tyrosine biosynthetic pathway. Inhibiting CM directly disrupts the bacteria’s ability to synthesize aromatic amino acids, resulting in cell death through “nutritional starvation” due to the depletion of essential metabolites. Humans obtain aromatic amino acids through diet and do not possess enzymes related to the shikimate pathway. Drugs targeting CM selectively disrupt bacterial metabolism without affecting host cell functions, thereby minimizing toxicity and significantly reducing the risk of adverse side effects. This makes CM a highly promising target for the development of novel anti-TB therapeutics. Seo et al. (2025), developed a novel TB subunit vaccine consisting of *Mycobacterium tuberculosis*-secreted chorismate mutase (TBCM) and a poly peptide derived from the hepatitis B virus (Poly6). The results showed that combining TBCM with Poly6 and the alum adjuvant significantly enhanced antigen-specific immune responses, effectively protecting mice from Mtb infection and substantially reducing bacterial load and lung inflammation. This combination TBCM vaccine demonstrated strong immunogenicity and therapeutic potential in mice, offering a new direction for the development of next-generation TB vaccines. Further optimization of the formulation and advancement of clinical translation research are needed in the future. Based on the 4-amino-1-methyl-3-propyl-1H-pyrazole-5-carboxamide fragment, Shukla et al. (2023), used a sonochemical synthesis method catalyzed by Wang resin to efficiently prepare novel pyrazole-pyrimidinone compounds. Among them, compounds 3b and 3c exhibited potent inhibition of Mtb CM and anti-TB activity, while also demonstrating low toxicity. These compounds are the first candidates to affect Mtb viability by inhibiting CM, providing an important foundation for the development of novel anti-TB drugs. Future research will focus on optimizing the structure and conducting *in vivo* studies.

### 2.4 Drug targets related to ferroptosis

**Figure 2:**
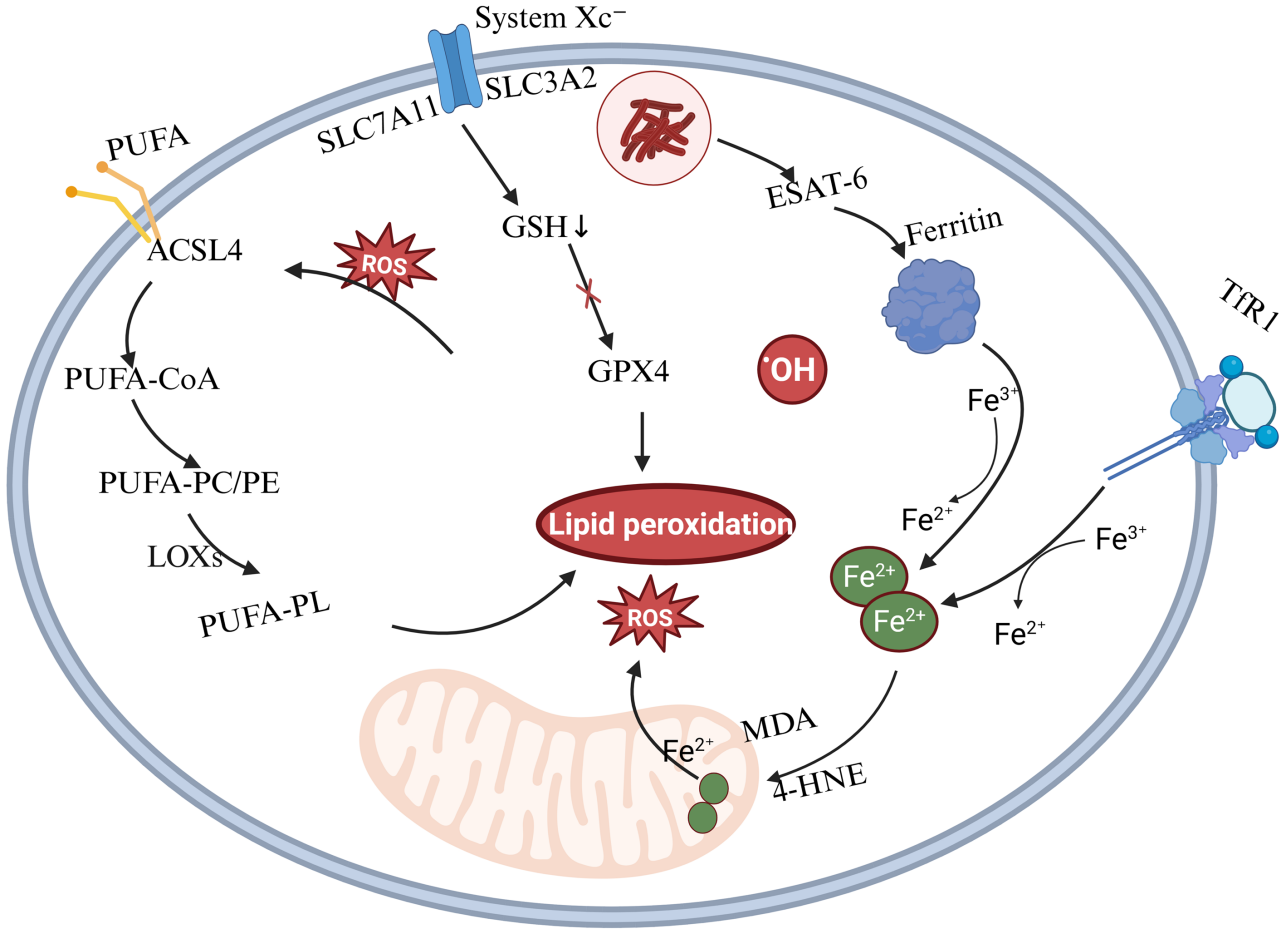
Mechanism of ferroptosis induced by Mtb infection. This figure illustrates the core molecular mechanisms of ferroptosis, a form of regulated cell death driven by iron-dependent lipid peroxidation. Three major pathways are involved: (1) Iron metabolism: Fe^3+^ enters cells via transferrin receptor 1 (TFR1) and is reduced to Fe^2+^, which promotes lipid peroxidation through hydroxyl radical generation via the Fenton reaction.; (2) Lipid peroxidation: Polyunsaturated fatty acids (PUFAs) in cell membranes undergo enzymatic oxidation by lipoxygenases (LOXs) or non-enzymatic peroxidation, resulting in the accumulation of toxic lipid reactive oxygen species (ROS) that trigger cell death; (3) Glutathione–GPX4 pathway: System Xc^−^ (SLC7A11) imports cystine, which is reduced to cysteine for glutathione (GSH) synthesis. GSH serves as a substrate for glutathione peroxidase 4 (GPX4), which reduces lipid hydroperoxides and inhibits ferroptosis.

Ferroptosis is a form of regulated cell death dependent on iron metabolism, characterized by elevated intracellular iron levels and intensified lipid peroxidation. After Mtb infection, the regulation of virulence factors such as ESAT-6 promotes the autophagy of ferritin, releasing iron ions and leading to the accumulation of free iron within cells, thereby intensifying oxidative damage. Additionally, Mtb infection diminishes the activity of the cysteine/glutamate transporter (System Xc^−^), leading to reduced synthesis of glutathione (GSH). This depletion of GSH subsequently inactivates glutathione peroxidase 4 (GPX4), impairing the clearance of lipid peroxides such as malondialdehyde (MDA) and 4-hydroxynonenal (4-HNE), thereby promoting cellular oxidative damage. Mtb enhances the expression of ACSL4, promoting the incorporation of polyunsaturated fatty acids (PUFAs) into cell membrane phospholipids. This incorporation generates substrates highly susceptible to oxidation, thereby initiating a lipid peroxidation chain reaction that exacerbates cellular damage (Jumabayi et al., 2024). The detailed mechanism of ferroptosis is shown in Figure 2.

#### 2.4.1 BACH1

BTB Domain and CNC Homolog 1 (BACH1) is a transcription factor that binds to specific sequences in the promoter region of the ferritin heavy chain gene, inhibiting ferritin gene expression and regulating iron storage and release. By regulating the ratio of ferritin heavy and light chains, BACH1 affects the structure and function of ferritin, leading to abnormal iron storage and release (Nishizawa et al., 2023).

Amaral et al. (2024), discovered that BACH1 is closely linked to disease progression during Mtb infection, suggesting its potential role in regulating the immune response and influencing pathological processes associated with the infection. *Bach1* mRNA expression of mononuclear cells in peripheral blood from TB patients was significantly higher compared to healthy controls and individuals with latent infection. In animal models, Mtb infection increases Bach1 expression in lung tissue, which is closely associated with necrotic areas. BACH1 inhibits Nrf2 activity, downregulating the expression of antioxidant genes and thereby affecting the host’s antioxidant defense system. In *Bach1* knockout mice, after Mtb infection, there was elevated *Gpx4* expression and decreased lipid peroxidation in lung tissue, indicating that BACH1 deficiency enhances the host’s antioxidant defense. Additionally, targeting BACH1 may represent a novel strategy to inhibit the progression of TB. Inhibiting BACH1 function has shown significant effects in reducing pathological damage and enhancing bacterial clearance, providing new targets and directions for TB treatment research.

#### 2.4.2 Mb3523c

The Mce4 family is a critical effector protein for Mycobacterium virulence, facilitating infection through the regulation of host cell death and immune evasion mechanisms. The Mb3523c protein from *Mycobacterium bovis* (*M. bovis*) belongs to the Mce4 family and shares 100% homology with the Rv3493c protein from Mtb H37Rv strain, suggesting functional similarities of their roles during infection. Mb3523c interacts with the Y237 and G241 of host HSP90 protein, stabilizing LAMP2A on the lysosomal membrane and promoting chaperone-mediated autophagy. This process leads to the degradation of GPX4, triggering ferroptosis and facilitating the transmission of *M. bovis*. Blocking the Mb3523c-HSP90 interaction or inhibiting the CMA pathway may reduce ferroptosis, potentially controlling the progression of TB (Wang et al., 2025). The interaction between Mb3523c and HSP90 provide new potential targets for TB therapy. Designing the inhibitors to block this interaction may effectively suppress bacterial virulence and transmission.

#### 2.4.3 Rv1324

The secretory protein Rv1324 has a strong homology with thioredoxins and may possess a thioredoxin function, enabling Mtb to defend against reactive oxygen species (ROS) and reactive nitrogen species (RNS) within the host (Wolfe et al., 2010). Research has shown that Rv1324 can enhance the persistence of Mtb by activating ferroptosis, which leads to pathological damage and inflammation in the lungs of mice. Rv1324, as a novel virulence factor of Mtb, promotes persistent infection and lung damage through a dual mechanism of antioxidant defense and induction of host ferroptosis. Targeting Rv1324 or the ferroptosis pathway may provide new therapeutic strategies for TB treatment (Shi et al., 2023).

## 3 Tuberculosis treatment strategies

### 3.1 Antimicrobial peptides

**Figure 3:**
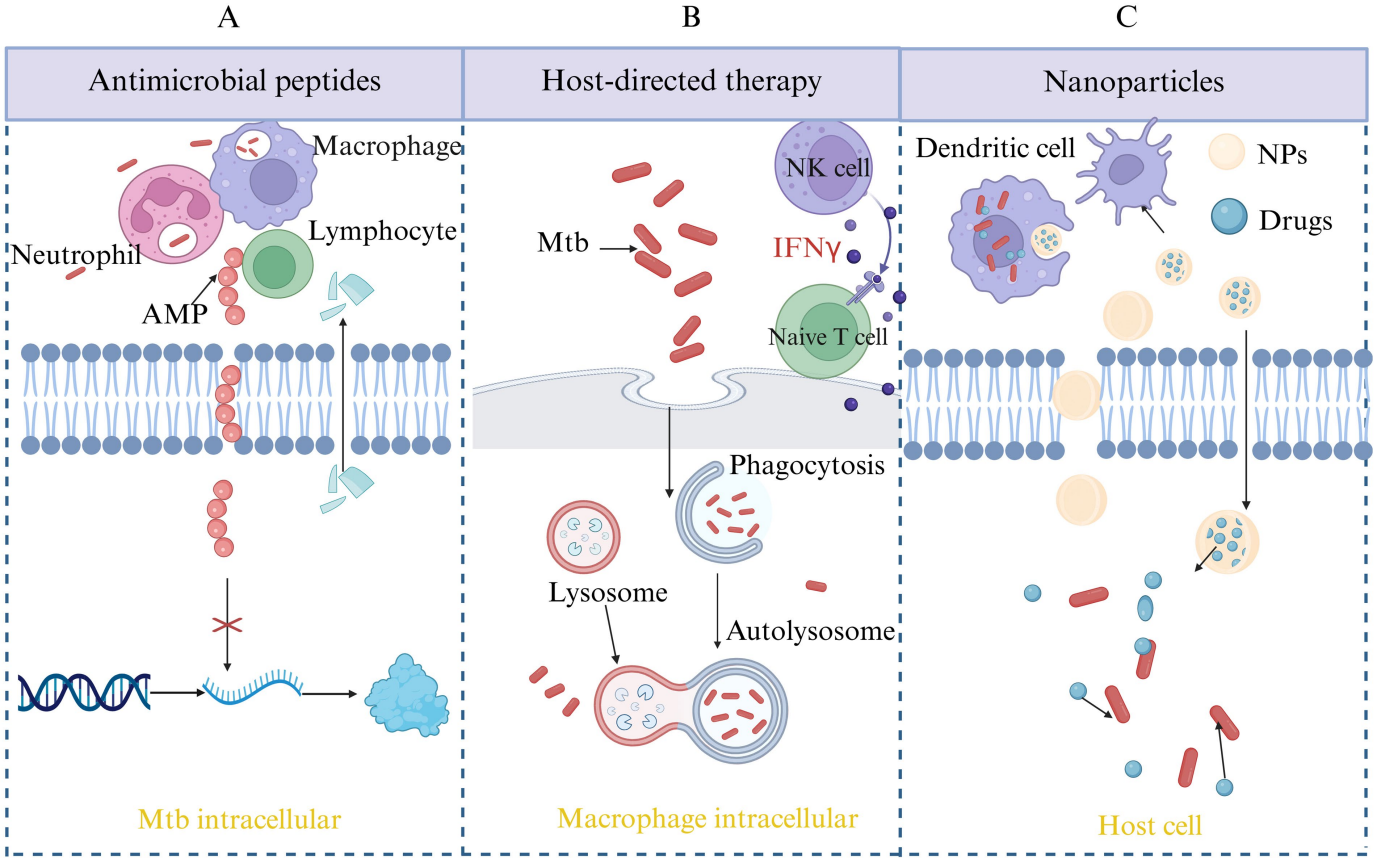
Novel therapeutic strategies for TB. This figure outlines emerging approaches in TB treatment beyond conventional antibiotics. **(A)** AMP therapy: AMPs exert direct bactericidal activity against Mtb by disrupting the bacterial membrane, modulating immune responses, and interfering with intracellular survival mechanisms. **(B)** Host-directed therapy (HDT): HDT aims to enhance the host’s immune defense or mitigate harmful inflammation during infection. Strategies include modulation of autophagy, inhibition of immune checkpoints, and targeting metabolic or inflammatory pathways to improve disease outcomes. **(C)** Nanoparticle-based delivery systems: Nanoparticles offer improved pharmacokinetics, targeted drug delivery, and enhanced bioavailability of anti-TB drugs or immunomodulators. This approach can reduce toxicity, overcome drug resistance, and improve treatment adherence.

Antimicrobial peptides (AMPs) are small molecules with various biological activities, including antibacterial, antiviral, and antifungal properties. They can be found in microorganisms, mammals and humans, and so on. Mtb has a lipid-rich, thick cell wall that makes it difficult for traditional antibiotics to penetrate. AMPs exist in a cationic form, enabling them to interact with the anionic components of bacterial cell membranes. This interaction disrupts the membrane’s integrity, causing leakage of intracellular contents and bacterial cell death, thereby overcoming the penetration barriers that limit the efficacy of traditional antibiotics. AMPs can penetrate bacterial cells, interfering with essential biosynthetic processes like protein synthesis, DNA replication, and RNA transcription which can inhibit bacterial growth, reproduction, and metabolic activity. Additionally, certain AMPs possess immunomodulatory properties that can stimulate the immune system of host, eliciting a targeted immune response against specific pathogens. The specific antimicrobial mechanisms are summarized and shown in Figure 3A. Due to their low cytotoxicity, AMPs are considered an effective alternative therapy for treating DR-TB, with promising potential for use in the treatment of MDR-TB (Mehta et al., 2022; Jacobo-Delgado et al., 2023).

Tenland et al. (2018), found that a novel derivative of the fungal antimicrobial peptide plectasin (Xiang et al., 2015), named NZX, exhibited bactericidal activity against Mtb. This non-toxic peptide can inhibit both clinical strains of Mtb and an MDR strain at therapeutic concentrations, with its therapeutic potential comparable to that of rifampicin. Shao et al. (2024), formulated a dry powder inhalation combination of the antimicrobial peptide D-ALK and isoniazid for the treatment of MDR-TB. The combination was also effective against Mtb resistant strains with mutations in KatG or InhA. In summary, the synergistic combination of INH and D-ALK peptide formulated as a dry powder inhaler provides a novel therapeutic approach for MDR-TB.

AMPs, with their unique bactericidal mechanisms, immunomodulatory functions, and low risk of resistance, show significant potential in the treatment of TB. Despite challenges related to stability, toxicity, and cost, AMPs are poised to become a key component of next-generation anti-TB therapies. They offer innovative solutions for global TB prevention and control through advancements in synthetic biology, nanotechnology, and immunology. Future research should focus on clinical transformation and the development of novel AMPs.

### 3.2 Host-directed therapy

Host-directed therapy (HDT) represents a departure from conventional antimicrobial treatments by focusing on activating and enhancing the host’s immune response. This strategy aims to improve the immune system’s ability to recognize, target, and eliminate pathogens while minimizing harm to the host’s own tissues, as shown in Figure 3B. This strategy not only addresses the limitations of traditional treatments but also offers the potential to reduce resistance development during therapy, thereby enhancing treatment efficacy and improving patient outcomes (Udinia et al., 2023; Jeong et al., 2022).

Mishra et al. (2023), investigated the anti-TB activity of soybean lectin (SBL) in differentiated THP-1 cells (dTHP-1) and elucidated its molecular mechanism, which involves cytokine-mediated autophagy. The study found that SBL treatment activated the PI3K/Akt/CREB signaling pathway, which in turn triggered the P2RX7-mediated pathway, resulting in a significant increase in IL-6 expression. The released IL-6 then interacted with IL-6R*α*, activating the JAK2/STAT3/Mcl-1 pathway, which further regulated autophagy and ultimately inhibited Mtb growth. Cytokine therapy and cytokine-mediated autophagy have become crucial host-directed therapeutic strategies for inhibiting Mtb growth in the host. Cytokine therapy boosts the host’s immune response to Mtb by modulating its immune system, while cytokine-mediated autophagy facilitates the intracellular autophagic process to eliminate the bacteria. The integration of these two strategies opens new possibilities and offers hope for enhancing host-mediated suppression of Mtb.

In recent years, significant progress has been made in the research of HDT drugs, with several compounds identified as potential HDT drugs, which enhance the host’s defense against pathogens by modulating the immune system. Sulfalazine targets the amino acid transporter system xc, reducing intracellular Mtb bacterial colonization and alleviating pulmonary lesions, making it a promising HDT drug for treating TB (Fu et al., 2024). Amiodarone is an HDT drug that exerts its anti-Mtb effect by inducing autophagy, thereby enhancing the host’s ability to control Mtb infection (Kilinç et al., 2024). Ursolic acid synergistically inhibits the Akt/mTOR and TNF-α/TNFR1 signaling pathways while promoting autophagy, thereby regulating macrophage pyroptosis and necroptosis. This makes it a promising adjunctive host-targeted drug for TB therapy (Shen et al., 2023). Berberine is an FDA-approved drug that effectively clears both drug-sensitive and drug-resistant Mtb by regulating ROS/Ca^2+^ to activate macrophage autophagy, making it a potential candidate for HDT (Zhang S. et al., 2023).

With the continuous development of HDT drug research, many drugs that have been approved for other diseases are being reused for TB HDT research. For example, imatinib promotes phagosome maturation, while vitamin D_3_ and sodium butyrate induce autophagy. Metformin and N-acetylcysteine regulate immune responses, and aspirin and statins modulate inflammation. These drugs have shown varying efficacy and mechanisms of action in clinical trials, the specific drugs are listed in Table 3.

**Table 3:** Repurposed drugs for the treatment of tuberculosis with host directed therapy.

FDA approved drugs	Clinical indications	Mechanism	Reference
Imatinib	Treatment of diverse hematologic malignancies and solid tumors	Accelerate and modulate immune responses, induce granulomatous lesions	Cleverley et al. (2023)
Metformin	Treatment of type 2 diabetes and cardiovascular protection	Enhance dehydroepiandrosterone synthesis while maintaining cortisol homeostasis; reduce the secretion of chemokines.	Gonzalez-Muñiz et al. (2024) and Pavan Kumar et al. (2024)
Vitamin D_3_	Promote calcium and phosphorus absorption to support bone health. Regulates the immune system	It promotes macrophage activation, enhancing their ability to phagocytose and kill Mtb; anti-inflammatory; induces autophagy	Reddy et al. (2024)
Phenylbutyrate and Vitamin D_3_	Regulates intestinal flora; enhanced immunomodulation; improvement of metabolic function	Accelerates clinical recovery, sputum culture conversion, induces macrophages to produce antimicrobial peptide LL-37	Rekha et al. (2018)
Aspirin	Antipyretic and analgesic, anti-inflammatory, anti-rheumatic, anti-platelet aggregation	All-cause death between inclusion and week 40; inhibition of platelet aggregation; modulate the inflammatory response	Maitre et al. (2022)
Aspirin and Ibuprofen	Antipyretic and analgesic, anti-inflammatory and anti-rheumatic	Inhibition of cyclooxygenase-1 and cyclooxygenase-2	Arias et al. (2023)
Ibuprofen	Antipyretic and analgesic, anti-inflammatory	Regulates macrophage polarization	Wang et al. (2024)
Dexamethasone	Anti-inflammatory, anti-shock, anti-tumor, anti-allergic and immunosuppressive effects	Immunomodulation, induce cell apoptosis	Lara-Espinosa et al. (2023)
Dexamethasone and ketoprofen	Anti-inflammatory, analgesic and antipyretic	Significantly reduced the increase in SFT and thus the number of positive responses in the SIT test	Ortega et al. (2022)
N-acetylcysteine	Anti-inflammatory, antioxidant, mucolytic, detoxification	Promoted glutathione synthesis and attenuated oxidative stress damage	Charalambous et al. (2024)
Doxycycline	Antibacterial and anti-inflammatory, malaria prevention	Bactericidal activity against MDR and XDR strains. As an MMP-1 inhibitor in the treatment of spondylitis TB	Zhang C. et al. (2023) and Siregar et al. (2023)
Fluvastatin	Lowering blood lipids and preventing cardiovascular diseases	Prompting monocytes/macrophages to become foamy cells and enhancing macrophage killing of Mtb	Montero-Vega et al. (2024)
Atorvastatin	Lowering blood lipids, treatment of coronary heart disease, anti-inflammatory and improve vascular endothelial function.	LAM activity against Mtb and improvement of drug permeability in granulomas	Davuluri et al. (2023)

### 3.3 Nanoparticles

Traditional treatments for TB face challenges such as low drug delivery efficiency, long treatment durations, significant side effects, and drug resistance. Nanoparticles (NPs) have the potential to address these issues by improving drug delivery, reducing side effects, and overcoming resistance. NPs can actively target infection sites, such as alveolar macrophages, to enhance drug concentration at the infection site. Additionally, they can encapsulate multiple drugs, providing a synergistic effect that helps inhibit drug-resistant mutations. Due to the biofilm formed by Mtb hindering drug penetration, the small size and surface charge modulation of NPs can enhance their ability to penetrate the biofilm barrier (Hetta et al., 2023). Nanoparticles can be classified into four types based on their material composition (as shown in Table 4), with the core therapeutic strategies focusing on targeted drug delivery systems and overcoming drug resistance, the specific mechanism is shown in Figure 3C.

**Table 4:** Classification and key characteristics of various nanoparticles.

Types	Characteristics
Metal nanoparticles	Photothermal/photodynamic effects, magnetic targeting
Polymeric nanoparticles	Controlled degradation, pH/enzyme responsive release
Liposome	Highly biocompatible, modifiable targeting ligands
Dendritic macromolecular nanoparticles	High drug loading capacity, surface functionalization

#### 3.3.1 Targeted delivery systems

Rifampicin is a core drug for the treatment of TB, but oral or injectable administration faces challenges such as low bioavailability, high systemic toxicity, and insufficient pulmonary targeting. NPs offer a potential solution, significantly enhancing the therapeutic efficacy of anti-TB drugs. Leite et al. (2025), developed a pulmonary inhalation delivery system, PN-PCG-RIF, designed to improve the administration of rifampicin directly to the lungs. The NPs loaded with rifampicin, using phthalated cashew gum (PCG) as the matrix, are embedded in micron-sized particles. This dual-scale nanoparticle-microparticle composite design enhances pulmonary deposition, reduces premature drug clearance, improves macrophage targeting and promotes efficient intracellular drug release. It enables targeted delivery through microparticle disintegration and nanoparticle acid-responsive drug release, facilitating efficient macrophage uptake within 6 h. The particles show good biocompatibility, with cell viability greater than 90% for both alveolar epithelial cells and macrophages. The particles, significantly enhanced the pulmonary delivery efficiency and antimicrobial activity of rifampicin through the innovative design of a nanoparticle-microparticle composite structure combined with natural polymer modification and pulmonary targeting strategies, offering vital experimental support for the development of inhalation therapies for TB. Gong et al. (2024), developed Polylactic-co-glycolic acid-polyethylene glycol (PLGA-PEG) NPs which was modified by Triantennary N-Acetylgalactosamine (Tri-GalNAc) loaded with the STING agonist SR717 and Mtb fusion protein TP, resulting in the TP/Tri-GalNAc-PLGA-PEG-SR717 (TP/GPS) formulation. This system effectively activates dendritic cells (DCs) and stimulates cellular immune responses, providing a novel approach for the development of subunit vaccines. *In vitro* and *in vivo* experiments demonstrated that Tri-GalNAc modification enhanced the targeting of NPs to DCs, while SR717 promoted DCs maturation and activation. TP/GPS can induce antigen-specific T cell immune responses and reduce pulmonary bacterial load. This study provides an innovative and effective adjuvant strategy for developing subunit vaccines targeting intracellular pathogens, with potential applications in vaccines and drug delivery for other diseases.

Nanoparticle-based targeted drug delivery systems can enhance drug efficacy, reduce side effects, and improve patient adherence. These systems have significant research potential and application prospects in TB treatment, enabling more efficient and precise drug delivery to target sites while minimizing systemic toxicity.

#### 3.3.2 Nanoparticles with anti-biofilm activity

The complex structure of the biofilm enhances bacterial tolerance to adverse environmental conditions, such as desiccation, extreme temperatures, or antibiotics, significantly improving bacterial survival. The formation of biofilms by Mtb is a key factor in its evasion of host immune responses and the development of antibiotic resistance (Muñoz-Egea et al., 2023). Traditional anti-TB drugs have low efficacy in eliminating bacteria within biofilms. With the increasing cases of multidrug-resistant bacteria, it is urgent need to develop effective treatments targeting biofilm-associated infections. Functionalized NPs offer a novel strategy to address this challenge by enhancing drug penetration, disrupting the extracellular matrix, and providing synergistic antibacterial effects.

Currently, the treatment of biofilm infections mainly relies on antibiotics. However, the antibiotic resistance of biofilms urgently calls for more efficient and innovative antimicrobial agents and biofilm-targeting strategies, such as the combination of antibiotics with biofilm-disrupting agents. Zhang Z. et al. (2023), developed a composite nanoparticle (CL@LEV-NPs) with PLGA as the shell, while cellulase (CL) and levofloxacin (LEV) acted as the core. After ultrasonic irradiation, the nanoparticles generate a significant amount of reactive oxygen species, facilitating their penetration to the biofilm. This enhances the effective drug concentration within the biofilm and significantly reduces drug resistance. CL@LEV-NPs exhibit a low hemolysis rate *in vitro* and do not cause liver or kidney dysfunction *in vivo*. These findings indicate that the combination of ultrasound and composite nanoparticles is a non-invasive, safe, and highly effective novel strategy for combating biofilm infections caused by Mtb.

2G0 is a novel polycationic dendrimer nanoparticle with excellent oral bioavailability and low toxicity, positioning it as a promising candidate for the treatment of Mtb infections (Mignani et al., 2021). It is found that 2G0 exhibits strong activity against Mtb, Nontuberculous Mycobacteria (NTM), and drug-resistant MAB, with efficacy comparable to meropenem. It can synergize with various antibiotics, including rifampin, bedaquiline, clofazimine, and linezolid, significantly reducing bacterial load, preventing the formation of drug-resistant mutants, and ensuring no bacterial relapse after combination therapy. By disrupting membrane structures and inhibiting resistant mutations, 2G0 offers a new strategy for treating MDR/XDR-TB and acts as a novel anti-mycobacterial drug or antibiotic delivery agent (Imran et al., 2024).

Nanoparticles enhance drug permeability and antimicrobial activity, effectively overcoming the resistance of Mtb biofilms and improving therapeutic outcomes. Future research should further explore the application of nanoparticles in biofilms and develop novel nanoparticle-based drug delivery systems to provide the new strategies and approaches for TB treatment.

## 4 Discussion

The widespread use and misuse of anti-TB drugs, along with the high transmissibility and latency of TB, it remains one of the most serious global threats to human health. In light of the growing prevalence and spread of drug-resistant strains, it is urgent to develop safer and more effective anti-TB therapies. In recent years, significant advancements have been made in the development of anti-TB drugs, particularly in identifying new drug targets and designing innovative therapies. The development of anti-TB drugs involves a wide range of targets, encompassing key steps in the growth and metabolism of Mtb. In addition to the targets discussed in this review, many new potential targets remain to be explored. For example, pathways involved in lipid synthesis, gene regulation, material transport, protein secretion, and host interactions with Mtb could serve as the important targets for the next generation of anti-TB drugs.

In light of the current challenges, future research on anti-TB drugs should focus on the several key directions: First, it is essential to strengthen the discovery and validation of new drug targets, particularly those involving unique metabolic pathways in Mtb. Second, the more selective and effective drug compounds should be developed. The explored strategies such as chemical synthesis, natural product screening, and drug structure optimization to enhance bioactivity, improve pharmacokinetics, and minimize side effects. Finally, optimizing drug design through computational models and systems biology is an efficient strategy. Computational simulations of drug-target binding interactions can predict drug resistance and tolerance, helping to mitigate the risk of failure early in drug design.

In recent years, novel anti-TB treatment strategies have emerged, overcoming the limitations of traditional therapies through the synergistic action of multiple mechanisms. AMPs target and disrupt the integrity of the mycobacterial cell membrane through their amphipathic structure, while also interfering with PG synthesis, inhibiting ribosomal function, and inducing DNA damage. Their multitarget mechanism significantly reduces the risk of resistance, although issues related to stability and *in vivo* degradation still need to be addressed. Host-directed therapies exert antimicrobial effects by modulating the host cell’s immune response and metabolic pathways, enhancing macrophage autophagy, and inhibiting signaling pathways that promote bacterial survival. While this approach offers the advantage of reduced resistance development, its clinical application still faces critical challenges, including the precision of target selection and the evaluation of potential side effects. Regulating ferroptosis-related pathways can enhance the host’s immune response to Mtb, but its safety and efficacy in vivo still require further investigation. NPs have become a key direction for anti-TB drug research, with their main advantages being improved drug targeting, reduced side effects, enhanced efficacy, and overcoming drug resistance. However, the in vivo metabolism and safety of NPs still require further optimization. While new anti-TB treatment strategies show promise, significant obstacles remain before they can be widely applied in clinical practice. Future research should focus on elucidating the molecular mechanisms of new strategies, optimizing treatment regimens to enhance efficacy and reduce side effects, and validating their effectiveness and safety through clinical trials.

Except the therapeutic targets and strategies mentioned above, vaccination either serve as an alternative intervention. Up to now, vaccination remains one of the most effective strategies for the long-term control of infectious diseases. BCG, a live attenuated vaccine derived from *M. bovis*, is currently the only approved vaccine for TB prevention. It provides substantial protection against severe forms of TB in children, such as miliary and meningeal TB, but offers limited efficacy in adults. Moreover, as a live vaccine, BCG may cause localized or disseminated infections in immunocompromised individuals (Lange et al., 2022). Consequently, the development of safer and more effective next-generation TB vaccines is a key priority in global TB control efforts.

TB vaccine candidates in development and clinical trials are broadly categorized according to their immunization strategy into three types: preventive replacement vaccines, booster preventive vaccines, and therapeutic vaccines. VPM1002 is a recombinant BCG strain in which the *ureC* gene has been replaced with the *Listeriolysin O* gene, leading to enhanced antigen presentation and improved immunogenicity. Preclinical studies in goat models have demonstrated that VPM1002 has a safety profile comparable to BCG (Figl et al., 2023). It is currently undergoing Phase III clinical trials (Singh et al., 2024). MTBVAC is an attenuated live strain of Mtb engineered through the deletion of two key virulence genes, *phoP* and *fadD26*. MTBVAC preserves the complete antigenic repertoire of Mtb, including RD1-encoded immunodominant antigens such as ESAT-6 and CFP-10, which are absent in BCG (Martín et al., 2021). Phase Ib–IIa clinical trials in adults demonstrated that MTBVAC exhibits a safety and reactogenicity profile comparable to BCG, while eliciting superior immunogenicity (Luabeya et al., 2025). Moreover, a neonatal trial conducted in South Africa showed that MTBVAC, at equivalent dosing, induced stronger immune responses with reduced reactogenicity compared to BCG. The vaccine is currently undergoing Phase III clinical evaluation in South Africa (Tameris et al., 2025).

M72/AS01E is a subunit vaccine composed of a recombinant fusion protein derived from two Mtb antigens, Mtb32A and Mtb39A, and formulated with the AS01 adjuvant. It is designed to boost immune responses following BCG vaccination and is primarily targeted at adolescents and adults (Wang et al., 2023). Studies have shown that M72/AS01E provides 54.0% protection against the progression of latent Mtb infection to pulmonary TB, with efficacy sustained for at least 3 years (Van Der Meeren et al., 2018; Tait et al., 2019). In 2025, people living with HIV were included in the ongoing global registration Phase III trial (Dagnew et al., 2025). H56: IC31 is a recombinant subunit vaccine composed of three Mtb antigens: Ag85B (Rv1886c), ESAT-6 (Rv3875), and Rv2660c. Similar in design to M72/AS01E, it is intended as a booster to BCG to enhance protective immunity against pulmonary TB (Tait et al., 2024). However, administration of H56: IC31 at the completion of TB treatment did not reduce the risk of disease relapse. While the vaccine is well tolerated and immunogenic, it may potentially increase the risk of relapse due to endogenous reactivation of latent Mtb strains (Borges et al., 2025).

RUTI is a therapeutic vaccine composed of inactivated Mtb cell wall nanofragments encapsulated in liposomes. It is designed as an adjunctive treatment for TB, primarily targeting latent TB infection (LTBI). By stimulating a robust Th1-type immune response, RUTI enhances the host’s ability to control Mtb (Cardona and Amat, 2006). It holds promise for both the prevention and immunotherapeutic management of TB (Vilaplana et al., 2011). Phase IIb clinical trials have demonstrated its potential to improve treatment outcomes and shorten the duration of therapy without inducing drug resistance (NCT05455112). However, definitive evidence from Phase III trials is still required to confirm its clinical utility. ID93 + GLA-SE is a recombinant subunit TB vaccine composed of a fusion protein (ID93) consisting of four Mtb antigens (Rv2608, Rv3619, Rv3620, and Rv1813), combined with the TLR-4 agonist adjuvant GLA-SE. Designed to enhance Th1-type immune responses against Mtb, it serves as a potential BCG booster with both preventive and therapeutic applications (Siddiqui et al., 2025). The vaccine has demonstrated a favorable safety profile in BCG-vaccinated healthy individuals (Choi et al., 2023). Phase IIa trial data also indicate that ID93 + GLA-SE is safe and immunogenic in patients with drug-sensitive pulmonary TB (Day et al., 2021). The recent results show that the thermostable single-vial formulation of ID93 + GLA-SE exhibits comparable safety and immunogenicity to the non-thermostable two-vial formulation, without negatively impacting clinical use. This simplifies distribution and reduces overall costs (Sagawa et al., 2023). However, the effectiveness of this vaccine in preventing TB relapse following chemotherapy remains to be fully validated in future studies.

Although the BCG vaccine offers some protection against severe TB in children, its effectiveness in adults is limited and it poses risks for immunocompromised individuals. New-generation TB vaccines are being developed with a focus on greater safety and efficacy. Several candidate vaccines have demonstrated favorable safety profiles and immunogenicity, but their protective efficacy still needs to be confirmed through phase III clinical trials. Looking ahead, advancing vaccine development and promoting global implementation will be crucial for controlling and ultimately eliminating TB.

In addition, except the traditional antibiotics, phages exhibit high specificity and can target drug-resistant strains without harming the host’s beneficial microbiota. As a potential alternative or adjunctive strategy for TB treatment, phage therapy is gaining increasing attention (Ouyang et al., 2023).

Several specific bacteriophages, including D29, TM4, and DS6A, have been shown to effectively lyse Mtb. These phages are capable of infecting and lysing Mtb even under simulated TB pathological conditions, including low pH, hypoxia, and intracellular environments (Jeyasankar et al., 2024). In a humanized mouse model infected with Mtb *H37Rv*, intravenous administration of phage DS6A significantly reduced bacterial loads in the lungs and spleen, improved body weight, and enhanced lung function, demonstrating therapeutic potential within a human-like immune context (Yang F. et al., 2024; Yang L. et al., 2024). In 2019, a British patient with a NTM infection following lung transplantation showed significant improvement after personalized phage therapy, following failure of standard antibiotics (Dedrick et al., 2019). In 2022, a 26-year-old with advanced cystic fibrosis and chronic *Mycobacterium abscessus* infection was similarly cured using phage treatment (Nick et al., 2022). Despite these encouraging cases, no clinical trials have been conducted for Mtb, and current evidence remains limited to individual NTM infections. These findings highlight the need to advance phage therapy toward clinical application in TB.

Phage therapy holds promising potential in overcoming drug resistance in TB treatment; however, its clinical application faces significant challenges. Firstly, due to the high host specificity of phages, personalized selection is required for different Mtb strains, emphasizing the urgent need to establish comprehensive phage libraries covering diverse clinical isolates, or to develop broad-host-range and genetically engineered phages. Secondly, limited delivery efficiency restricts therapeutic efficacy, as phages face challenges penetrating barriers like macrophages and granulomas. Thus, targeted pulmonary delivery approaches, including aerosol inhalation and liposomal encapsulation, are essential to enhance phage bioavailability and treatment outcomes. Thirdly, phages are rapidly cleared by the host immune system *in vivo* and exhibit limited stability; prolonged use may also drive the emergence of phage-resistant bacterial mutants. Phage cocktails or combination of phage therapy with anti-TB drugs may mitigate resistance development and enhance therapeutic efficacy. Moreover, systematic clinical trial data and safety evaluations remain lacking. Future research should focus on engineering optimized phages, improving delivery systems, developing combination therapies, and establishing clear translational pathways to ensure the safe and effective application of phage therapy in TB treatment.

Although considerable efforts have been made, TB remains a serious global health threat, particularly with the rise of multidrug-resistant and extensively drug-resistant strains. To address these challenges, future anti-TB drug research must be driven by multidisciplinary innovation and collaborative strategies. Continued exploration of novel targets, optimization of drug structures, and application of advanced technologies will be essential for the development of more effective and durable therapies. Our work will provide an important theoretical basis and methodological support for the innovative development of anti-TB drugs, which will be benefit for prevention and effective treatment of TB in the future.

## Footnotes

^1^ http://zincpharmer.csb.pitt.edu/pharmer.html
