# Supplementary material for: Progress of anti-tuberculosis drug targets and novel therapeutic strategies
Source: Front Microbiol. 2025 Sep 5;16:1637254. doi: 10.3389/fmicb.2025.1637254 (PMC12446298; doi:10.3389/fmicb.2025.1637254)
Supplement: Supplementary file 1 [file Data_Sheet_1.PDF]

Supplementary Material

Yang Zhang[1], Ruiying Wu[1], Mingrui Sun[1], Xiaotian Li[1], Ren Fang[1], Jiayin Xing[1], Zhaoli Li[3*], Yurong Wen[2*], Ningning Song[1*]

[1] Weifang Key Laboratory of Respiratory Tract Pathogens and Drug Therapy, School of Life Science and Technology, Shandong Second Medical University, Weifang 261053, China

[2] The First Affiliated Hospital of Xi'an Jiaotong University, Xian 710049, China

[3] SAFE Pharmaceutical Technology Co., Ltd., Beijing 100000, China

*** Correspondence:**
Zhaoli Li: lizhaoli@safeglp.com

Yurong Wen: Yurong.Wen@xjtu.edu.cn

Ningning Song: songningning@sdsmu.edu.cn

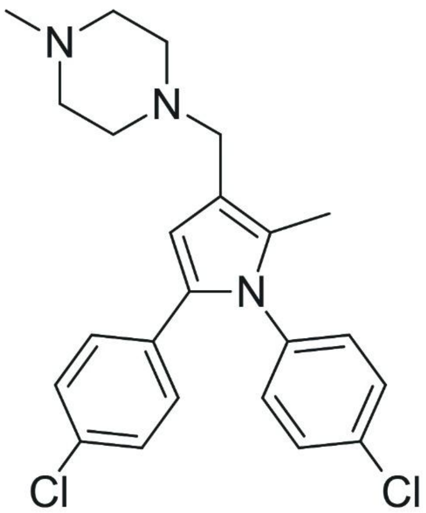

Supplementary Figure 1. Chemical structure of BM212

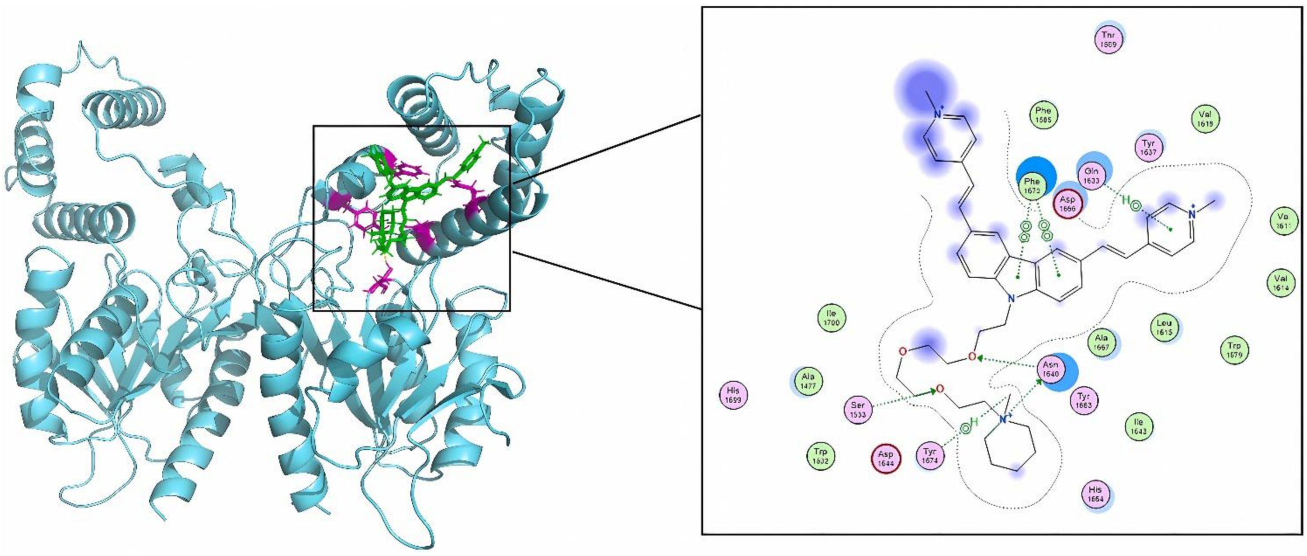

Supplementary Figure 2. Molecular docking of Pks13 and BMVC-8C3O.

Adapted from Liu et al.,2025. https://doi.org/10.1016/j.tube.2024.102579

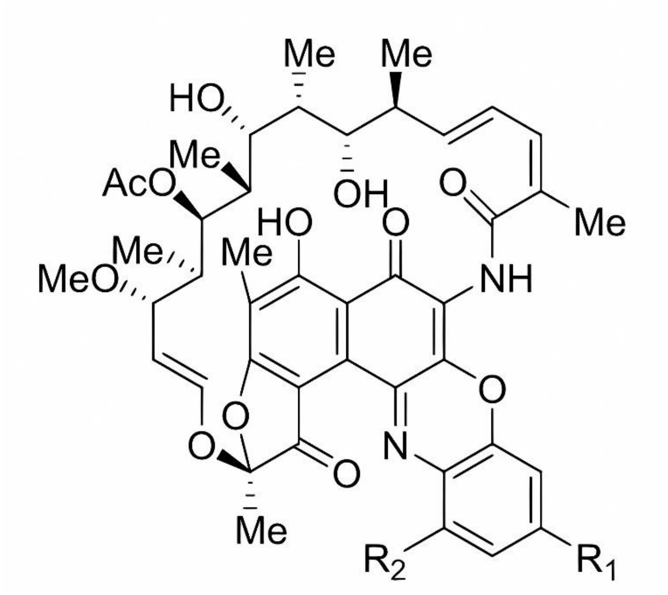

Supplementary Figure 3. Chemical structure of bxRIF

Adapted from Rajeswaran et al.,2022. https://doi.org/10.1021/acsinfecdis.1c00636
